# Enzalutamide Induces Apoptotic Insults to Human Drug-Resistant and -Sensitive Glioblastoma Cells via an Intrinsic Bax-Mitochondrion-Cytochrome C Caspase Cascade Activation Pathway

**DOI:** 10.3390/molecules27196666

**Published:** 2022-10-07

**Authors:** Chia-Yu Chang, Jui-Tai Chen, Tso-Hsiao Chen, Ruei-Ming Chen

**Affiliations:** 1 Department of Neurology, Chi Mei Medical Center, Tainan 71004, Taiwan; 2 Center for General Education, Southern Taiwan University of Science and Technology, Tainan 71005, Taiwan; 3 Cell Physiology and Molecular Image Research Center, Wan Fang Hospital, Taipei Medical University, Taipei 11696, Taiwan; 4 Department of Anesthesiology, Shuang Ho Hospital, Taipei Medical University, Taipei 23561, Taiwan; 5 Department of Anesthesiology, School of Medicine, College of Medicine, Taipei Medical University, Taipei 11031, Taiwan; 6 Division of Nephrology, Department of Internal Medicine, Wan Fang Hospital, Taipei Medical University, Taipei 11696, Taiwan; 7 Graduate Institute of Medical Sciences, College of Medicine, Taipei Medical University, Taipei 11031, Taiwan; 8 International Ph.D. Program for Cell Therapy and Regeneration Medicine, College of Medicine, Taipei Medical University, Taipei 11031, Taiwan; 9 Anesthesiology and Health Policy Research Center, Taipei Medical University Hospital, Taipei 11031, Taiwan; 10 TMU Research Center of Cancer Translational Medicine, Taipei Medical University, Taipei 11031, Taiwan

**Keywords:** glioblastoma multiforme, androgen receptor, enzalutamide, apoptosis, intrinsic apoptotic mechanism

## Abstract

Glioblastoma multiforme (GBM) is the most common and malignant brain tumor. Temozolomide (TMZ) is the first-line chemotherapeutic drug for treating GBM. However, drug resistance is still a challenging issue in GBM therapy. Our preliminary results showed upregulation of *androgen receptor* (*AR*) gene expression in human GBM tissues. This study was designed to evaluate the effects of enzalutamide, a specific inhibitor of the AR, on killing drug-resistant and -sensitive glioblastoma cells and the possible mechanisms. Data mining from The Cancer Genome Atlas (TCGA) database revealed upregulation of *AR* messenger (m)RNA and protein expressions in human GBM tissues, especially in male patients, compared to normal human brains. In addition, expressions of *AR* mRNA and protein in human TMZ-sensitive U87 MG and -resistant U87 MG-R glioblastoma cells were elevated compared to normal human astrocytes. Exposure of human U87 MG and U87 MG-R cells to enzalutamide concentration- and time-dependently decreased cell viability. As to the mechanism, enzalutamide killed these two types of glioblastoma cells via an apoptotic mechanism. Specifically, exposure to enzalutamide augmented enzyme activities of caspase-9 rather than those of caspase-8. Moreover, enzalutamide successively triggered an elevation in levels of the proapoptotic Bax protein, a reduction in the mitochondrial membrane potential, release of cytochrome c, cascade activation of caspases-3 and -6, DNA fragmentation, and cell apoptosis in human TMZ-sensitive and -resistant glioblastoma cells. Pretreatment with Z-VEID-FMK, an inhibitor of caspase-6, caused significant attenuations in enzalutamide-induced morphological shrinkage, DNA damage, and apoptotic death. Taken together, this study showed that enzalutamide could significantly induce apoptotic insults to human drug-resistant and -sensitive glioblastoma cells via an intrinsic Bax-mitochondrion-cytochrome c-caspase cascade activation pathway. Enzalutamide has the potential to be a drug candidate for treating GBM by targeting the AR signaling axis.

## 1. Introduction

Glioblastoma multiforme (GBM) is the most common and deadliest primary brain tumor [1]. The average 5-year mortality for GBM is more than 90%. The causes explaining the malignance and recurrence of GBM are very multifaceted [2]. One of the critical reasons is the location where GBM occurs. GBM usually occurs in the brain, so it is extremely difficult for neurosurgeons to completely eliminate entire tumors. Unfortunately, residual glioblastoma cells can rapidly proliferate, migrate, invade, and recur at other sites of the brain [1,3]. Another critical point elucidating the poor prognosis of GBM patients is that glioblastoma cells can straightforwardly escape from targeted drugs and radiation therapies [4]. Temozolomide (TMZ), a DNA-alkylating agent, is the main, first-line chemotherapeutic drug for treating GBM [1]. Inappropriately, more than 50% of GBM patients may be initially TMZ-resistant or eventually develop drug resistance during the process of antitumor therapy [5]. All of these multifaceted reasons consequently result in the poor prognoses of GBM patients. The average survival time for GBM patients whose brain tumors are removed and treated with subsequent concurrent chemo- and radiotherapy (CCRT) is about 15 months [4]. To the present, the mechanisms explaining the transformation of normal glial cells to glioblastoma cells and subsequent tumorigenesis of GBM are not well known. Multiple molecules and signaling alliances were recently found, such as inhibition of O^6^-methylguanine-DNA methyltransferase (MGMT) and the bradykinin (BK)-BK B1 receptor axis, which is involved in oncogenesis, malignance, and recurrence of GBM [5,6]. Exploring all of those mechanisms would definitely be beneficial for creating de novo therapeutic strategies for treating GBM patients.

The androgen receptor (AR), encoded by the *NR3C4* gene, is a ligand-binding nuclear transcription factor that belongs to the steroid hormone nuclear receptor family [7]. There are three major functional domains, including an N-terminal transcriptional regulation domain, a DNA-binding domain, and a ligand-binding domain, that exist in the structure of the AR. Among them, the DNA-binding domain is the most highly conserved and contains two zinc fingers that specifically recognize the DNA consensus 5′-GGA/TACANNNTGTTCT-3′ sequence, named the AR-DNA-binding element. Testosterone and its main metabolite, 5α-dihydrotestosterone, are two chief androgen ligands of the AR protein [8]. After binding to these androgen ligands, the dimerized AR can be translocated from the cytoplasm to nuclei for specific regulation of thousands of target genes involved in cell differentiation and proliferation [7]. The androgen-AR signaling axis is closely associated with the development and maintenance of reproductive, cardiovascular, hemopoietic, musculoskeletal, immune, and neural systems [9]. Furthermore, the AR signaling alliance plays a critical role in prostate development. Nonetheless, the deregulation of AR-mediated signaling events in the prostate can also trigger the initiation, promotion, and progression of prostate cancer [10]. In addition, testosterone-AR-transducing signals may contribute to oncogenesis and tumorigenesis of other malignances, including breast, ovary, bladder, lung, liver, and kidney cancers [11]. Recently, steroid hormones of estrogen and androgen were reported to possibly be involved in the development of glioblastomas [12]. Thus, the roles of the AR in tumorigenesis of glioblastomas have attracted a lot of research in wide-ranging investigations [13]. Moreover, targeting AR signaling was suggested to have the potential for treating many types of cancer, especially prostate cancer [10,13]. As a result, dysregulation of AR-mediated signals may be an important factor in the development of many tumors and cancers. In contrast, silencing of AR signals can potentially be applied as a new therapeutic approach for numerous cancers besides prostate cancer.

Enzalutamide (C_21_H_16_F_4_N_4_O_2_S), a non-steroid antiandrogen, is a potent oral inhibitor of the AR [14]. Characteristically, enzalutamide is broadly used to treat prostate cancer. In particular, enzalutamide can be effectively administered to patients with metastatic castrate-resistant prostate cancer [15]. As to the mechanisms, levels of the AR in prostate cancer patients are usually upregulated compared to those of normal human prostate tissues. Moreover, expression of the *AR* gene in castration-resistant prostate cancer is induced compared to castration-sensitive types [16]. The augmented AR can be activated by androgens via an autocrine mechanism, and this triggers the expression of certain genes involved in tumorigenesis, therapy resistance, and tumor recurrence. In contrast, administration of enzalutamide can attenuate activation of the AR by preventing translocation of the AR from the cytoplasm to nuclei. Compared to female GBM patients, a greater incidence and worse outcomes were detected in male patients [17]. Hence, the sex hormone may affect the malignance of GBM. Furthermore, Rodríguez-Lozano et al. showed that treatment of glioblastoma cells with testosterone caused significant enhancements in cell proliferation, migration, and invasion [17]. Lastly, treatment with enzalutamide reduced the stemness of GBM stem cells [18]. In human glioblastomas, levels of testosterone are augmented [19]. Our previous study also showed upregulation of *AR* gene expression in human GBM tissues compared to normal human brains [20]. Thus, targeting the testosterone-AR axis can possibly be used to treat human GBM. However, the effects of enzalutamide on human drug-sensitive and -resistant glioblastoma cells are little known. Therefore, this study was designed to evaluate the killing of human TMZ-sensitive and -resistant glioblastoma cells by enzalutamide and the possible mechanisms.

## 2. Results

### 2.1. Upregulation of AR mRNA and Protein Expressions in GBM Patients

**Figure 1:**
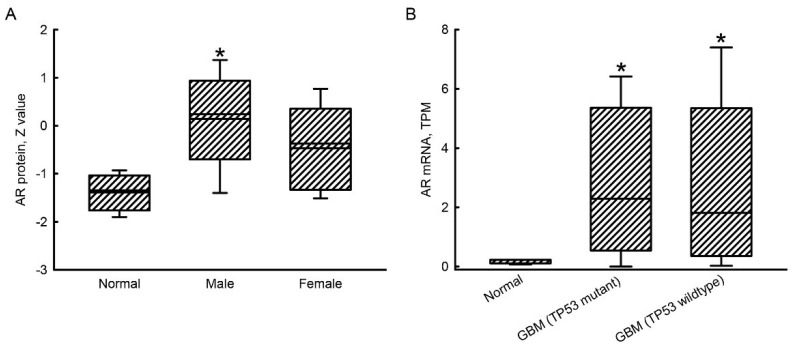
Upragulation of androgen receptor (AR) mRNA and protein expressions in human glioblastoma multiforme (GBM) patients. Levels of the AR protein in normal human brains (*n* = 10) and human male (*n* = 55) and female (*n* = 44) GBM tissues were mined from The Cancer Genome Atlas (TCGA) database and analyzed using the UALCAN platform (http://ualcan.path.uab.edu/, accessed on 12 July 2022) (**A**). Corelations of AR mRNA expression in human GBM tissues with wild-type and mutant tumor protein 53 (TP53) were carried out in the UALCAN system (**B**). Each value represents the minimum, lower quartile, median, upper quartile, and maximum. An asterisk (*) indicates that the value significantly differs from the respective normal group, at *p* < 0.05. TPM, transcripts per million.

Compared to normal human brains, levels of the AR protein in human male GBM tissues were enhanced by 3-fold (Figure 1A). In contrast, there was no significant difference in levels of the AR protein between female GBM tissues and normal brains. In human GBM patients with TP53 mutant and wild types, respective expressions of the *AR* gene were induced by 4.2- and 3.0-fold (Figure 1B).

### 2.2. Exposure to Enzalutamide Decreased Viabilities of TMZ-Sensitive and -Resistant Glioblastoma Cells

**Figure 2:**
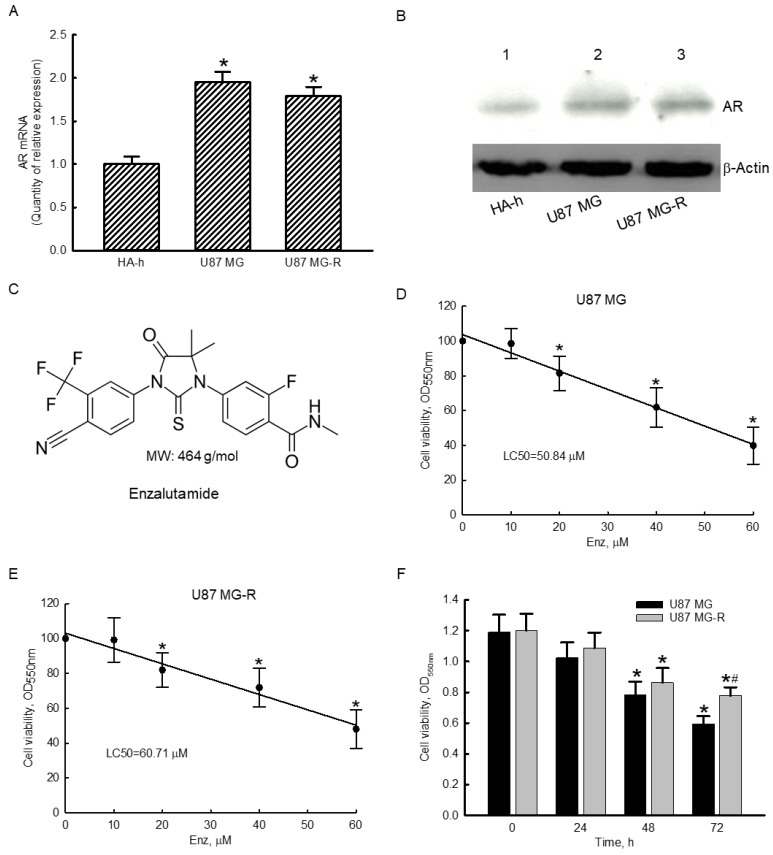
Effects of enzalutamide (Enz) on viabilities of human temozolomide (TMZ)-sensitive U87 MG and TMZ-resistant U87 MG-R glioblastoma cells. Expressions of androgen receptor (AR) mRNA in human HA-h astrocytes, and U87 MG and U87 MG-R glioblastoma cells were measured using a real-time PCR (**A**). Levels of AR were immunodetected ((**B**), top panel). β-Actin was analyzed as the internal control (bottom panel). The chemical structure of Enz and its molecular weight are shown (**C**). Human U87 MG (**D**) and U87 MG-R (**E**) glioblastoma cells were exposed to 10, 20, 40, and 60 μM Enz for 72 h. Cell viability was analyzed using a colorimetric method. The 50% lethal concentrations (LC_50_s) of enzalutamide in human U87 MG and U87 MG-R glioblastoma cells were then calculated. Human U87 MG and U87 MG-R glioblastoma cells were treated with 50 μM Enz for 24, 48, and 72 h, and cell viability was assayed (**F**). Each value represents the mean ± SEM for *n* = 6. The symbols * and # respectively indicate that the values significantly (*p* < 0.05) differed from the control and U87 MG groups.

Compared to normal human HA-h astrocytes, expressions of AR mRNA in human TMZ-sensitive U87 MG and TMZ-resistant U87 MG-R glioblastoma cells were elevated by 95% and 79%, respectively (Figure 2A). Compared to HA-h cells, levels of AR protein in human U87 MG and U87 MG-R glioblastoma cells were upregulated (Figure 2B). The chemical structure of enzalutamide is shown in Figure 2C. Its molecular weight is 464 g/mol. Exposure of human TMZ-sensitive U87 MG glioblastoma cells to 10 μM enzalutamide for 72 h did not affect cell viability (Figure 2D). However, when the concentration of enzalutamide reached 20 μM, the viability of U87 MG glioblastoma cells decreased by 18%. Exposure to enzalutamide at 40 and 60 μM led to significant 38% and 60% respective reductions in the viability of human drug-sensitive U87 MG cells. The 50% lethal concentration (LC_50_) of enzalutamide toward human TMZ-sensitive U87 MG glioblastoma cells was 50.84 μM (Figure 2D). Treatment of human drug-resistant U87 MG-R glioblastoma cells with 10 μM enzalutamide for 72 h did not influence cell viability (Figure 2E). After exposure to enzalutamide at 20, 40, and 60 μM for 72 h, viabilities of human U87 MG-R glioblastoma cells significantly declined by 18%, 28%, and 52%, respectively. The LC_50_ of enzalutamide toward human TMZ-resistant U87 MG-R glioblastoma cells was 60.71 μM (Figure 2E). Moreover, exposure of human U87 MG and U87 MG-R cells to 50 μM enzalutamide for 24 h did not change cell viabilities (Figure 2F). After treatment for 48 h, enzalutamide at 50 μM decreased the viabilities of human U87 MG and U87 MG-R glioblastoma cells by 34% and 28%, respectively. Exposure of human U87 MG and U87 MG-R cells to enzalutamide at 50 μM for 72 h caused 50% and 35% reductions in cell viabilities, respectively (Figure 2F). In comparison, treatment with 50 μM enzalutamide for 72 h led to more insults to human drug-sensitive U87 MG glioblastoma cells than to drug-resistant U87 MG-R cells (Figure 2F).

### 2.3. Enzalutamide Significantly Induced DNA Frgmentation and Cell Apoptosis in Human TMZ-Sensitive and -Resistant Glioblastoma Cells

**Figure 3:**
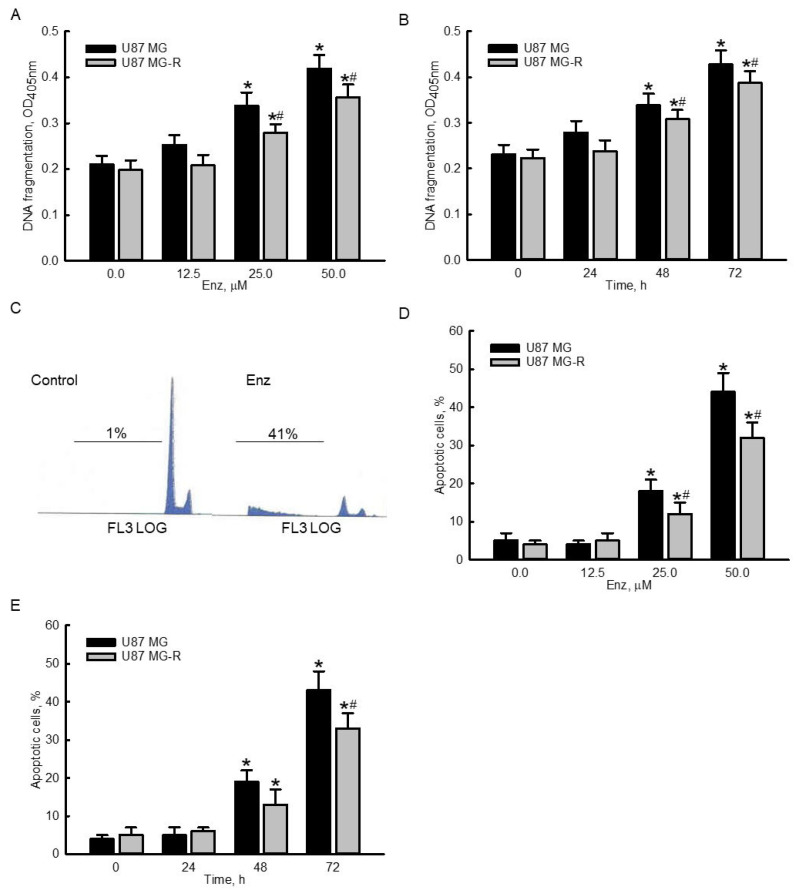
Enzalutamide (Enz) induced DNA fragmenation and cell apoptosis in human temozolomide (TMZ)-sensitive U87 MG and TMZ-resistant U87 MG-R glioblastoma cells. Human U87 MG and U87 MG-R glioblastoma cells were treated with 12.5, 25, and 50 μM Enz for 72 h (**A**,**D**) or with 50 μM enzalutamide for 24, 48, and 72 h (**B**,**E**). DNA fragmentation was determined using an ELISA method (**A**,**B**). Apoptotic cells were quantified using flow cytometry (**C**–**E**). Representative histograms of FACS analyses in human U87 MG cells are shown (**C**). Each value represents the mean ± SEM for *n* = 6. The symbols * and # respectively indicate that the values significantly (*p* < 0.05) differed from the control and U87 MG groups.

Exposure of human TMZ-sensitive U87 MG and TMZ-resistant U87 MG-R glioblastoma cells to 12.5 μM for 72 h did not trigger DNA fragmentation (Figure 3A). In contrast, when the concentration reached 25 μM, enzalutamide induced DNA fragmentation in human U87 MG and U87 MG-R cells by 61% and 41%, respectively. Treatment with 50 μM enzalutamide for 72 h led to 100% and 80% DNA fragmentation in human U87 MG and U87 MG-R cells, respectively (Figure 3A). Furthermore, treatment of human U87 MG and U87 MG-R glioblastoma cells with enzalutamide for 24 h did not affect DNA fragmentation (Figure 3B). After exposure for 48 and 72 h, enzalutamide at 50 μM induced 46% and 85% DNA fragmentation in human U87 MG cells and 38% and 74% DNA breakage in human U87 MG-R cells, respectively (Figure 3B). Representative histograms of fluorescence-activated cell sorting (FACS) analyses showed that exposure to 50 μM enzalutamide for 72 h increased the proportions of human U87 MG cells at the sub-G1 phase (Figure 3C). Exposure to 12.5 μM enzalutamide for 72 h did not induce apoptosis of human drug-sensitive or -resistant glioblastoma cells (Figure 3D). Enzalutamide at 25 and 50 μM significantly induced apoptosis of human U87 MG and U87 MG-R glioblastoma cells by 18% and 40% in U87 MG as well as 12% and 32% in U87 MG-R glioblastoma cells, respectively. Exposure of human drug-sensitive and -resistant glioblastoma cells to 50 μM enzalutamide for 48 and 72 h caused time-dependent apoptotic insults (Figure 3E). Interestingly, enzalutamide induced more DNA apoptosis insults in human TMZ-sensitive U87 MG cells than in TMZ-resistant U87 MG-R cells (Figure 3A,B,D,E).

### 2.4. Enzalutamide Specifically Elevated Caspase-9 Activities and Proapoptotic Bcl-2-Associated X Protein (Bax) Levels but Decreased the Mitochondrial Membrane Potential (MMP) in Human TMZ-Sensitive and -Resistant Glioblastoma Cells

**Figure 4:**
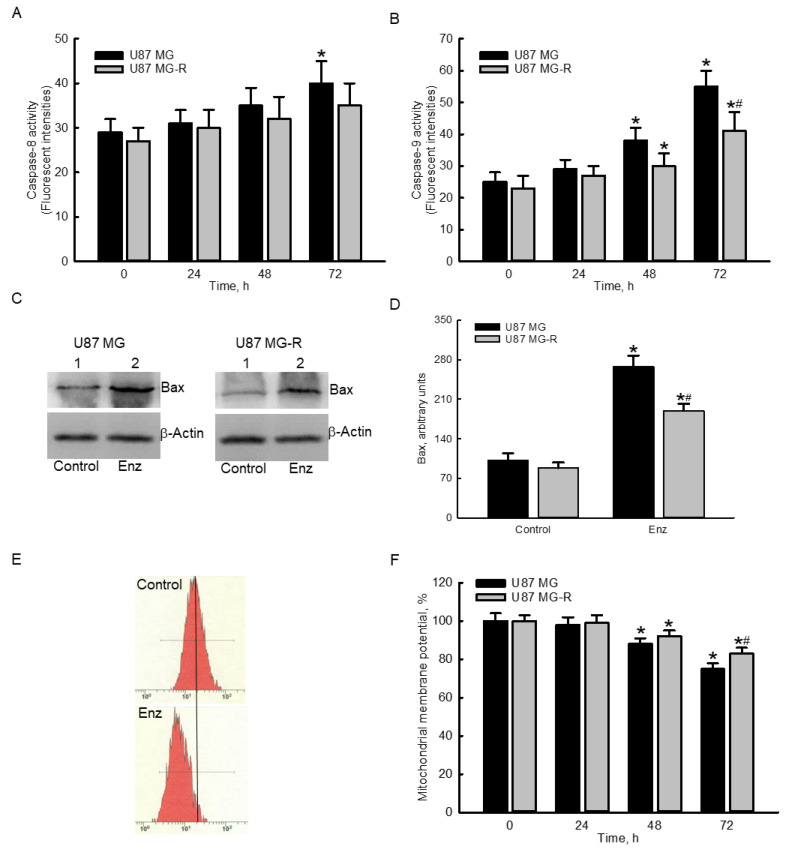
Enzalutamide (Enz) specifically activated caspase-9, increased cellular Bax levels, and decreased the mitochondrial membrane potential in human temozolomide (TMZ)-sensitive U87 MG and TMZ-resistant U87 MG-R glioblastoma cells. Human U87 MG and U87 MG-R glioblastoma cells were treated with 50 μM Enz for 24, 48, and 72 h. Activities of capase-8 (**A**) and -9 (**B**) were assayed using fluorogenic substrate methods. Levels of the proapoptotic Bax in human U87 MG and U87 MG-R glioblastoma cells were immunodetected ((**C**), top panels). β-Actin was analyzed as the internal control (bottom panels). These protein bands were quantified and statistically analyzed (**D**). The mitochondrial membrane potentials of human U87 MG and U87- MG-R cells were measured using flow cytometry (**E**,**F**). Representative histograms of FACS analyses in human U87 MG cells are shown (**E**). Each value represents the mean ± SEM for *n* = 6. The symbols * and # respectively indicate that the values significantly (*p* < 0.05) differed from the control and U87 MG groups.

Exposure of human TMZ-sensitive U87 MG glioblastoma cells to 50 μM enzalutamide for 24 and 48 h did not change the activity of caspase-8 (Figure 4A). However, following treatment for 72 h, enzalutamide at 50 μM caused a significant 38% increase in caspase 8 activity in human U87 MG cells. In contrast, exposure to 50 μM enzalutamide for 24, 48, and 72 h did not influence activity of caspase-8 in human drug-resistant glioblastoma cells (Figure 4A). After treatment with 50 μM enzalutamide for 24 h, activities of caspase-8 in both human U87 MG and U87 MG-R glioblastoma cells were not altered (Figure 4B). In contrast, when the time periods reached 48 and 72 h, enzalutamide at 50 μM significantly encreased caspase-8 activities by 52% and 120% in human U87 MG cells and 30% and 78% in human U87 MG-R cells, respectively (Figure 4B). Likewise, enzalutamide triggered more activation of caspase-9 in human U87 MG glioblastoma cells than in human U87 MG-R cells (Figure 4B). Moreover, treatment with 50 μM enzalutamide for 72 h augmented levels of the proapoptotic Bax protein in human U87 MG and U87 MG-R glioblastoma cells (Figure 4C, top panels). β-Actin was immunodetected as the internal control (bottom panels). These protein bands were quantified and statistically analyzed (Figure 4D). Exposure to enzalutamide for 72 h caused significant 110% and 101% increases in levels of the Bax protein in human U87 MG and U87 MG-R cells, respectively. Representative histograms of FACS analyses showed that exposure to 50 μM enzalutamide of human U87 MG cells for 72 h led to an obvious lessening (left shift) of the MMP (Figure 4E). Treatment of human U87 MG and U87 MG-R glioblastoma cells with 50 μM enzalutamide for 24 h did not affect the MMP (Figure 4F). However, when the treatment time period reached 48 and 72 h, enzalutamide decreased the MMP by 12% and 25% in human drug-sensitive U87 MG cells and 8% and 17% in human drug-resistant U87 MG-R cells, respectively. Similarly, enzalutamide more strongly reduced the MMP in human U87 MG than in human U87 MG-R glioblastoma cells (Figure 4F).

### 2.5. Enzalutamide Augmented Cytochrome c Levels and Subsequently Stimulated Cascade Activation of Caspases-3 and -6 in Human TMZ-Sensitive and TMZ-Resistant Glioblastoma Cells

**Figure 5:**
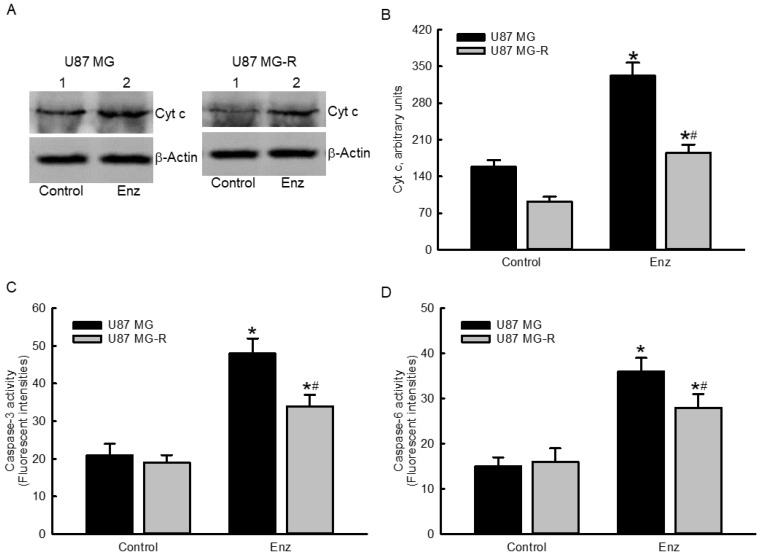
Enzalutamide (Enz) triggered cytochrome c (Cyt c) release and subsequent activation of caspases-3 and -6 in human temozolomide (TMZ)-sensitive U87 MG and TMZ-resistant U87 MG-R glioblastoma cells. Human U87 MG and U87 MG-R glioblastoma cells were treated with 50 μM Enz for 72 h. Levels of Cyt c in human U87 MG and U87 MG-R glioblastoma cells were immunodetected ((**A**), top panels). β-Actin was analyzed as the internal control (bottom panels). These protein bands were quantified and statistically analyzed (**B**). Activities of capase-3 (**C**) and -6 (**D**) were assayed using fluorogenic substrate methods. Each value represents the mean ± SEM for *n* = 6. The symbols * and # respectively indicate that the values significantly (*p* < 0.05) differed from the control and U87 MG groups.

Levels of cytochrome c in human TMZ-sensitive U87 MG and TMZ-resistant U87 MG-R glioblastoma cells were enhanced following exposure to 50 μM enzalutamide for 72 h (Figure 5A, top panels). Amounts of β-actin were measured as the internal control (bottom panels). All of these protein bands were quantified and statistically analyzed (Figure 5B). Enzaluatamide enhanced cytochrome c release by 2.1- and 1.8-fold in human U87 MG and U87 MG-R glioblastoma cells, respectively. Subsequently, exposure of human U87 MG and U87 MG-R glioblastoma cells to 50 μM enzalutamide led to 132% and 79% enhancements in activities of caspase-3, respectively (Figure 5C). Meanwhile, treatment with enzalutamide at 50 μM for 72 h led to 140% and 75% cascade activation of caspase-6 in human U87 MG and U87 MG-R glioblastoma cells, respectively (Figure 5D).

### 2.6. Suppressing Caspase-6 Activity Concurrently Attenuated Enzalutamide-Induced Morphological Changes, DNA Fragmentation, and Cell Apoptosis in Human TMZ-Sensitive and -Resistant Glioblastoma Cells

**Figure 6:**
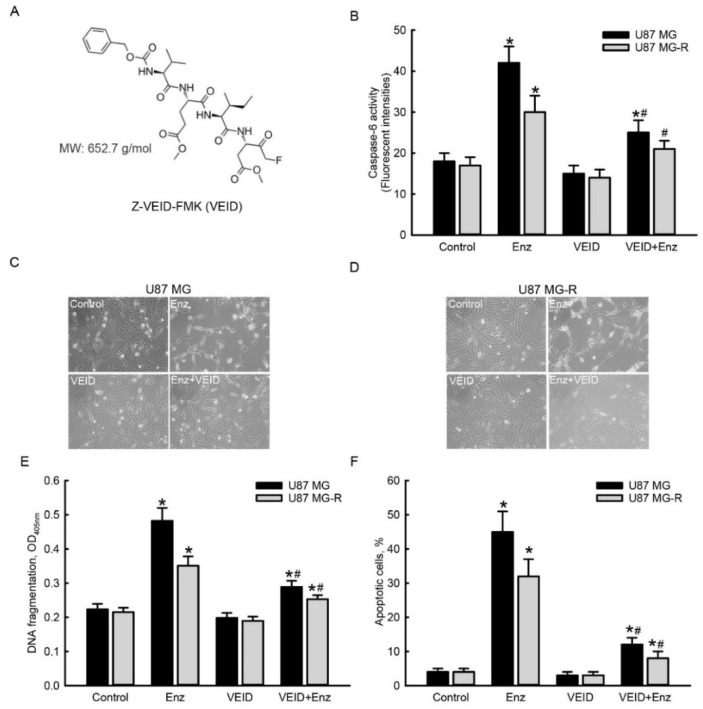
Suppressing caspase-6 activity concurrently attenuated enzalutamide (Enz)-induced DNA fragmentation and apoptotic death of temozolomide (TMZ)-sensitive U87 MG and TMZ-resistant U87 MG-R glioblastoma cells. The chemical structure of Z-VEID-FMK (VEID), a specific inhibitor of caspase-6, with a molecular weight of 652.7 g/mol is shown in (**A**). Human U87 MG and U87 MG-R glioblastoma cells were pretreated with 50 μM VEID for 1 h and then exposed to 50 μM Enz for 72 h. The activity of caspase-6 was assayed using a fluorogenic substract method (**B**). Cell morphologies were observed and photographed using a microscope (**C**,**D**). DNA fragmentation was quantified using an ELISA method (**E**). Apoptotic cells were analyzed using flow cytometry (**F**). Each value represents the mean ± SEM for *n* = 6. The symbols * and # respectively indicate that the values significantly (*p* < 0.05) differed from the control and Enz-treated groups.

The structure of Z-VEID-FM, a specific inhibitor of caspase-6, is shown in Figure 6A. The molecular weight of Z-VEID-FM is 652.7 g/mol. Exposure to 50 μM enzalutamide, respectively, enhanced caspase-6 activities by 133% and 76% in human TMZ-sensitive U87 MG and TMZ-resistant U87 MG-R glioblastoma cells (Figure 6B). Pretreatment with Z-VEID-FM at 50 μM did not affect the activities of caspase-6 in human U87 MG or U87 MG-R glioblastoma cells. However, pretreatment with Z-VEID-FM significantly attenuated enzalutamide-induced activation of caspase-6 in human drug-sensitive and -resistant glioblastoma cells by 75% and 100%, respectively (Figure 6B). Analysis of the cell morphology further showed that exposure to 50 μM enzalutamide for 72 h decreased cell numbers and induced cell shrinkage in human U87 MG cells (Figure 6C). Pretreatment with Z-VEID-FM did not change the cell morphology but obviously prevented enzalutamide-induced cell insults. Similar as human U87 MG glioblastoma cells, pretreatment with Z-VEID-FM did not affect the morphologies of human U87 MG-R cells but protected against enzalutamide-induced cell injury (Figure 6D). Furthermore, the enzalutamide-induced DNA fragmentation of human U87 MG and U87 MG-R glioblastoma cells meaningfully declined by 74% and 71%, respectively (Figure 6E). In addition, Z-VEID-FM did not trigger apoptosis of human U87 MG or U87 MG-R glioblastoma cells (Figure 6F). In contrast, pretreatment with Z-VEID-FM caused significant 73% and 75% attenuations, respectively, in enzalutamide-induced apoptotic insults to human drug-sensitive U87 MG and -resistant U87 MG-R glioblastoma cells.

## 3. Discussion

Enzalutamide can significantly kill human TMZ-sensitive and TMZ-resistant glioblastoma cells. Our data mining results showed upregulation of *AR* gene expression in human GBM patients, especially in male ones, compared to normal human brains. After binding to specific androgens, activated AR can be translocated from the cytoplasm to nuclei, where it regulates certain targeting genes [7]. Our present results further revealed that levels of *AR* mRNA in TMZ-sensitive U87 MG and TMZ-resistant U87 MG-R glioblastoma cells were significantly higher than in normal human astrocytes. Enzalutamide, a non-steroid inhibitor of AR activation, can suppress the translocation of this nuclear transcription factor to nuclei [14]. Treatment of human U87 MG and U87 MG-R glioblastoma cells with enzalutamide decreased cell viabilities in concentration- and time-dependent manners. GBM is the most common and aggressive brain tumor with very poor prognoses. TMZ is a first-line chemotherapeutic agent for treating GBM [4]. However, drug resistance is still a major challenge for GBM patients. In this study, we showed that exposure to enzalutamide caused a significant diminution in the viabilities of human TMZ-sensitive and -resistant glioblastoma cells. In prostate cancer, enzalutamide was shown to kill cancer cells by decreasing AR activity [21]. Our present study also demonstrated that enzalutamide could kill human drug-sensitive and -resistant glioblastoma cells. Accordingly, enzalutamide has the potential to be a drug candidate for GBM therapy.

The AR signaling pathway contributes to enzalutamide-induced insults to human TMZ-sensitive and -resistant glioblastoma cells. Our data mining present results from TCGA revealed that expressions of *AR* mRNA and protein in human GBM tissues were upregulated compared to normal human brains. In addition, expressions of AR mRNA and protein in human TMZ-sensitive U87 MG and -resistant U987 MG-R glioblastoma cells were upregulated compared to normal human astrocytes. Testosterone, synthesized by the testes and secreted to other tissues via the circulatory system, is the major androgen. A previous study showed upregulation of serum testosterone in male and female GBM patients [22]. Our unpublished data further demonstrated the effects of testosterone on inducing *AR* mRNA and protein levels in human drug-sensitive and -resistant glioblastoma cells. Thus, the upregulation of *AR* gene expression in GBM patients is due to the augmentation of testosterone. Schwartzbaum et al. reported that men more frequently suffer from GBM compared to women at a ratio of 3:2 [23]. The differences in levels of testosterone and its metabolites may lead to diverse incidences of GBM in male and female populations. The androgen/AR signaling contributes to the regulation of tumorigenesis, including stimulation of cell metabolism, proliferation, migration, and invasion [24,25]. Targeting AR signaling has been widely applied for the therapy of prostate cancer [13,25,26]. In various types of human glioblastoma cell lines, testosterone and 5α-dihydrotestosterone could promote cell proliferation, migration, and invasion, but flutamide, an antagonist of AR, blocked such an induction [9,17]. Orozco et al. also demonstrated that a combined treatment of dutasteride, an inhibitor of 5α-reductase, and flutamide had better effects to suppress metabolism, proliferation, and invasion of glioblastoma cells [27]. Besides, silencing the AR using RNA interference induced the death of human glioblastoma cells [28]. In the present study, we also showed the toxic effects of enzalutamide, an inhibitor of the AR, on killing human glioblastoma cells. Drug resistance is still a challenging issue in GBM research and therapy [5]. Our results revealed that enzalutamide significantly reduced the viability of human drug-resistant glioblastoma cells. Therefore, the testosterone-AR signaling axis can contribute to tumorigenesis, malignance, and drug resistance of GBM.

Enzalutamide can kill human TMZ-sensitive and -resistant glioblastoma cells specifically via an apoptotic mechanism. In parallel with declining cell viability, treatment with enzalutamide induced DNA fragmentation and subsequent cell-cycle arrest at the sub-G_1_ phase in concentration- and time-dependent manners. DNA fragmentation and cell-cycle arrest at the sub-G_1_ phases are two typical characteristics indicating that cells are undergoing apoptosis [29]. In addition, several lines of evidence, including activation and translocation of proapoptotic Bax from the cytoplasm to mitochondria, cytochrome c release from mitochondria to the cytoplasm, and cascade activation of caspases-9, -3, and -6, also showed that the enzalutamide-induced death mechanism in human drug-sensitive and -resistant glioblastoma cells occurred through an apoptotic pathway. Compared to normal astrocytes, glioblastoma cells are more defensive against/sensitive to apoptotic targeting [30]. Besides dysregulating heat shock protein and suppressing AR expression in prostate cancer cells, enzalutamide can directly induce cell apoptosis [31]. This study further demonstrated the effects of enzalutamide on apoptotic insults toward human glioblastoma cells. In general, drug resistance elevates the tolerance of glioblastoma cells to apoptotic injury [32]. Fascinatingly, enzalutamide was also able to induce apoptosis of human TMZ-resistant glioblastoma cells. An estimated 50~80% of GBM patients are drug-resistant [5]. Therefore, the administration of enzalutamide can overcome drug resistance-induced malignance of glioblastomas.

Enzalutamide induced apoptosis of human glioblastoma cells mainly via an intrinsic pathway. There are two distinct intrinsic mitochondrion-dependent and extrinsic Fas ligand/death receptor-dependent mechanisms that contribute to the regulation of cell apoptosis [33]. Enzymatic activation of caspases-8 and -9 is usually measured in order to determine if the extrinsic or intrinsic apoptotic mechanism is involved [34,35]. In the present study, treatment of human TMZ-sensitive glioblastoma cells with enzalutamide caused much greater activation of caspase-9 than caspase-8. Additionally, enzalutamide triggered the subsequent loss of the MMP and release of cytochrome c from mitochondria to the cytoplasm in human glioblastoma cells. As a result, enzalutamide induced apoptosis of human TMZ-sensitive glioblastoma cells via an intrinsic caspase-9-dependent mechanism. Similarly, enzalutamide also specifically increased caspase-9 activities in TMZ-resistant glioblastoma cells. Accordingly, enzalutamide induced apoptotic insults to human TMZ-sensitive and -resistant glioblastoma cells via an intrinsic pathway. Enzalutamide enhanced caspase-9 activation and induced cell apoptosis in human prostate LNCap cells [36]. In contrast, in enzalutamide-resistant prostate cancer cells, the activities of caspase-8 were upregulated [37]. Nevertheless, our present study showed that exposure to enzalutamide specifically increased the activities of caspase-9 in human drug-sensitive and -resistant glioblastoma cells.

Enzalutamide triggered a series of mitochondrial events in human glioblastoma cells. After exposure to enzalutamide, levels of Bax in human TMZ-sensitive and -resistant glioblastoma cells increased. Bax, a proapoptotic protein belonging to the Bcl-2 family, participates in cell apoptosis by translocation to the outer membranes of mitochondria [38]. Overproduction of Bax interrupts the balance with antiapoptotic proteins such as Bcl-2 and Bcl-XL and then drives cells to undergo programmed cell death. The accumulation of Bax in the outer membranes of mitochondria disrupts the membrane integrity [39]. Evidence indicating Bax-induced permeabilization of the mitochondrial membrane in human glioblastoma cells was the reduction of the MMP following exposure to enzalutamide. A previous study also showed that enzalutamide induced Bax expression in prostate cancer cells and triggered cell apoptosis [31]. In parallel with the enzalutamide-induced decrease in the MMP, levels of cytochrome c were concurrently augmented in human TMZ-sensitive and -resistant glioblastoma cells. Cytochrome c is a heme protein localized in the compartment between the inner and outer membranes of mitochondria [40]. The release of cytochrome c can potentially be used as an effective biomarker of mitochondrial and apoptotic damage. This study proved that enzalutamide induced sequential apoptotic events in human glioblastoma cells, including upregulation and translocation of Bax protein, loss of the MMP, and release of cytochrome c. Mitochondrial injury is a hallmark of cells undergoing intrinsic apoptosis [41]. Therefore, enzalutamide triggers apoptosis of human drug-sensitive and -resistant glioblastoma cells via an intrinsic mitochondrial mechanism.

Enzalutamide induced cascade activation of caspases-9, -3, and -6 in human glioblastoma cells. Treatment of human glioblastoma cells with enzalutamide stimulated the release of cytochrome c from mitochondria to the cytoplasm. Cytosolic cytochrome c can bind to apoptotic protease-activating factor (Apaf)-1, and the complex of cytochrome c-Apaf-1 consequently activates caspase-9 [42]. Our present results further showed that enzalutamide subsequently stimulated cascade activation of caspases-3 and -6. Caspase-3 is a downstream target of caspase-9 [43]. Hence, enzalutamide enhanced caspase-3 activity in human glioblastoma cells through stimulating the cytochrome c-mediated activation of caspase-9. Activated caspase-3 can subsequently cleave caspase-6, which then triggers its enzymatic activity [44]. In the present study, the enzalutamide-induced augmentation of caspase-6 activity was due to the activation of upstream caspases-9 and -3. Activated caspases-3 and -6 can then degrade key proteins, such as cytoskeletal and DNA repair proteins, leading to cell apoptosis [44]. Interestingly, suppressing caspase-6 activity with its specific inhibitor concurrently protected human drug-sensitive and -resistant glioblastoma cells from enzalutamide-induced morphological shrinkage, DNA fragmentation, and apoptotic insults. So, the enzalutamide-triggered cascade activation of caspases-9, -3, and -6 contributed to the apoptosis of human glioblastoma cells.

## 4. Materials and Methods

### 4.1. Data Mining

OMICS data of human GBM patients were mined from The Cancer Genome Atlas (TCGA) database (https://www.cancer.gov/about-nci/organization/ccg/research/structural-genomics/tcga, with data downloaded on 12 July 2022). Mined data were further analyzed using a tool of the UALCAN database system (http://ualcan.path.uab.edu, with data downloaded on 12 July 2022) as previously described [45]. In the database, the expression of *AR* mRNA and protein in normal human brains and GBM tissues were searched and analyzed.

### 4.2. Culture of Human Normal Astrocytes and Glioblastoma Cells

In this study, normal human Ha-h astrocytes purchased from ScienCell Research Laboratories (Carlsbad, CA, USA) and human U87 MG glioblastoma cells purchased from American Type Culture Collection (Manassas, VA, USA) were used as the experimental models. Human Ha-h astrocytes were cultured using a specific astrocyte medium (ScienCell Research Laboratories, Carlsbad, CA, USA). U87 MG glioblastoma cells were grown in Dulbecco’s modified Eagle’s medium (DMEM; Gibco-BRL Life Technologies, Grand Island, NY, USA) supplemented with 10% fetal bovine serum (FBS), l-glutamine (2 mM), penicillin (100 IU/mL), streptomycin (100 mg/mL), sodium pyruvate (1 mM), and nonessential amino acids (1 mM) at 37 °C in a humidified atmosphere of 5% CO_2_ as described previously [46]. Human Ha-h and U87 MG drug-sensitive glioblastoma cells were grown to confluence before drug treatment.

### 4.3. Preparation of Human Drug-Resistant Glioblastoma Cells

Human temozolomide (TMZ)-resistant U87 MG-R glioblastoma cells were selected from TMZ-sensitive U87 MZ cells according to our previous study [47]. TMZ was purchased from Sigma-Aldrich (St. Louis, MO, USA), and its purity was more than 98%. TMZ was freshly prepared by dissolving it in dimethyl sulfoxide (DMSO). Human TMZ-sensitive U87-MG cells (10^5^ cells) were seeded in 12-well tissue culture plates. U87 MZ glioblastoma cells were treated with 50 μM TMZ for 48 h. After drug treatment, U87 MG cells were harvested, diluted, and then subcultured in DMEM supplemented with 100 μM TMZ. The TMZ-resistant glioblastoma cell colony, named human drug-resistant U87 MZ-R cells, was trypsinized and subcultured.

### 4.4. Drug Treatment

Enzalutamide was bought from Sigma Aldrich and its purity was more than 98%. Enzalutamide was freshly dissolved in DMSO. Human U87 MG and U87 MG-R glioblastoma cells were treated with enzalutamide at 10, 20, 40, and 60 μM or at 12.5, 25, and 50 μM for 24, 48, and 72 h, and cell viability and apoptotic events were examined. Control cells received DMSO only. The concentration of DMSO was less than 0.1%.

### 4.5. Assays of Cell Morphology and Cell Viability

The toxicity of enzalutamide to human TMZ-sensitive and -resistant glioblastoma cells was assayed by determining the cell viability. Viabilities of human U87 MG and U87 MG-R glioblastoma cells were analyzed using a colorimetric method as described previously [48]. After drug treatment, human U87 MG and U87 MG-R glioblastoma cells (10^4^ cells) were seeded in DMEM with 3-(4,5-dimethylthiazol-2-yl)-2,5-diphenyltetrazolium bromide (MTT) at 0.5 mg/mL for 4 h. The medium was removed, and DMSO was added to dissolve the blue formazan products in human glioblastoma cells. The blue color was spectrophotometrically measured at an optical density (OD) of 550 nm. The 50% lethal concentrations (LC_50_s) of enzalutamide to human U87 MG and U87 MG-R were then calculated.

### 4.6. Quantification of DNA Fragmentation

Effects of enzalutamide on the integrity of chromosomal DNA in human glioblastoma cells were quantified using an enzyme-linked immunosorbent assay (ELISA) kit purchased from Boehringer Mannheim (Indianapolis, IN, USA) as described previously [49]. Human U87 MG and U87 MG-R glioblastoma cells (2 × 10^5^) were seeded in DMEM containing 5-bromo-20-deoxyuridine (BrdU) in 24-well tissue culture plates overnight. Then, cells were trypsinized and suspended. The cell suspension (100 μL) was cultured in 96-well tissue culture plates overnight. Then, human TMZ-sensitive and -resistant glioblastoma cells were treated with enzalutamide at 12.5, 25, and 50 μM for 24, 48, and 72 h in a CO_2_ incubator. After drug treatment, the BrdU-labeled DNA in the cytoplasm of human glioblastoma cells was measured using an Anthos 2010 microplate photometer (Anthos Labtec Instruments, Lagerhausstrasse, Wals/Salzburg, Austria) at a wavelength of 450 nm.

### 4.7. Analysis of Apoptotic Cells

Effects of enzalutamide on apoptotic insults to human TMZ-resistant U87 MG and -sensitive U87 MG-R glioblastoma cells were determined using a flow cytometric method as described previously [50]. Briefly, human glioblastoma cells (10^5^ cells) were seeded in 12-well tissue culture dishes overnight. Cells were exposed to 12.5, 25, and 50 μM enzalutamide for 24, 48, and 72 h. After drug treatment, human glioblastoma cells were trypsinized and fixed in ice-cold 80% ethanol. Fixed cells were centrifuged, washed with phosphate-buffered saline (PBS, 0.14 M NaCl, 2.6 mM KCl, 8 mM Na_2_HPO_4_, and 1.5 mM KH_2_PO_4_), and stained with propidium iodide (PI). Apoptotic cells were examined using a flow cytometer (Beckman Coulter, Fullerton, CA, USA) on the basis of a 560-nm dichromic mirror and a 600-nm bandpass filter. Representative histograms of FACS are shown, and proportions of apoptotic cells were then calculated and statistically analyzed.

### 4.8. Determination of the Intrinsic or Extrinsic Apoptotic Pathway

The pathway of enzalutamide-induced apoptotic insults to human TMZ-sensitive U87MG and -resistant U87 MG-R glioblastoma cells was determined by analyzing the activities of caspases-8 and -9 as described previously [51]. Briefly, human U87 MG and U87 MG-R glioblastoma cells were exposed to 50 μM enzalutamide for 24, 48, and 72 h. Following drug treatment, cell lysates were prepared. Protein concentrations of cell lysates were measured using a bicinchonic acid protein assay kit (Thermo Fisher Scientific, San Jose, CA, USA). Activities of caspases-8 and -9 were measured using the metabolites of their specific peptide substrates IETD and LEHD, respectively, that were conjugated with 7-amino-4-trifluoromethyl coumarin to detect the fluorescent intensities. Cell lysates at 25 mg were incubated with 50 mM of these two fluorogenic IETD and LEHD substrates in a cell-free system buffer (200 μL). Activities of caspases-8 and -9 were determined by measuring their fluorescent intensities with a spectrophotometer.

### 4.9. Immunodetection of AR, Bax and Cytochrome c Proteins

Levels of the AR, proapoptotic Bax, and cytochrome c proteins were immunodetected in order to determine the mechanisms of enzalutamide-induced apoptosis of human TMZ-sensitive U87 MG and -resistant U87 MG-R glioblastoma cells as described previously [51]. Human U87 MG and U87 MG-R glioblastoma cells were treated with 50 μM enzalutamide for 72 h. Then, cells were washed with PBS, and cell lysates were prepared by lysing human glioblastoma cells with an ice-cold lysis buffer containing 25 mM HEPES, 1.5% Triton X-100, 0.1% sodium dodecylsulfate (SDS), 0.5 M NaCl, 5 mM EDTA, and 0.1 mM sodium deoxycholate. A protease inhibitor cocktail of 10 mg/mL leupeptin, 0.27 U/mL aprotinin, and 100 mM phenylmethylsulfonyl fluoride (PMSF) was used to avoid protein degradation. Concentrations of cell lysates were measured with a bicinchonic acid protein assay kit (Thermo Fisher Scientific). Cell lysates (100 μg) were loaded in SDS-polyacrylamide gels and electrically separated. The separated proteins were then transferred to nitrocellulose membranes. Levels of AR, Bax, cytochrome c, and β-actin proteins on the same membrane were immunodetected. To avoid protein loss during striping, we cut the membrane into 3–4 strips based on the molecular weights of the protein markers shown. Then, these strips were individually used for immunodetection of AR, proapoptotic Bax, cytochrome c, and β-actin, respectively. AR, Bax, and cytochrome c were analyzed using mouse monoclonal antibodies against human AR and Bax (Santa Cruz Biotechnology, Santa Cruz, CA, USA) and pigeon cytochrome c protein (BioSource, Camarillo, CA, USA). β-Actin was detected using a mouse monoclonal antibody against mouse β-actin, purchased from Sigma-Aldrich, as an internal control. Immunoreactive proteins were detected with an enhanced chemiluminescence reagent (PerkinElmer, Waltham, MA, USA). The intensities of these protein bands were quantified using a digital analyzer (Syngene, Cambridge, UK) and densitometric software (Syngene). The detailed Western Blot images are shown in Figures S1–S3 (Supplementary Materials). 

### 4.10. Quantification of the Mitochondrial Membrane Potential (MMP)

The potential of mitochondrial membranes in human TMZ-sensitive U87 MG and -resistant U87 MG-R glioblastoma cells was quantified according to a method in a previous study [52]. Human U87 MG and U87 MG-R glioblastoma cells (5 × 10^5^ cells) were subcultured in 12-well tissue culture plates overnight. Then, glioblastoma cells were exposed to enzalutamide at 50 μM for 24, 48, and 72 h. After drug treatment, both types of these human glioblastoma cells were harvested into centrifuge tubes. An intracellular green-fluorescent probe, DiOC6, with a positive charge that can specifically accumulate in mitochondria, was added to the tube to reactivate the suspended cells at 37 °C for 30 min in a humidified atmosphere of 5% CO_2_. After the reaction, cells were spun down, and cell pellets were suspended in 1× PBS. Human glioblastoma cells with fluorescent DiOC6 were analyzed using flow cytometry (Beckman Coulter). Representative histograms of FACS are shown, and the fluorescent intensities were calculated and statistically analyzed.

### 4.11. Assay of Caspase-3 and -6 Activities

Cascade activation of caspases-3 and -6 in human TMZ-sensitive U87 MG and -resistant U87 MG-R glioblastoma cells was assayed using a fluorogenic substrate method as described previously [53]. After exposure to enzalutamide, the specific substrates, DEVD and VEID, were used to respectively measure activities of caspases-3 and -6. These fluorogenic substrates conjugated with 7-amino-4-trifluoromethyl coumarin for fluorescent detection were purchased from R&D Systems (Minneapolis, MN, USA). Cell lysates of human glioblastoma cells were prepared by lysing them in an ice-cold lysis buffer. Protein concentrations of cell lysates were measured using a bicinchonic acid protein assay kit (Thermo Fisher Scientific). Cell lysates at 25 mg total protein were mixed with 50 mM of the DEVD and VEID substrates in a cell-free system buffer at a volume of 20 μL. Activities of caspases-3 and -6 were measured with a fluorescent spectrophotometer.

### 4.12. Suppression of Caspase-6 Activation

A loss-of-function strategy by suppressing caspase-6 enzyme activity was used to determine the roles of caspase-6 in enzalutamide-induced apoptotic insults to human TMZ-sensitive U87 MG and -resistant U87 MG-R glioblastoma cells as described previously [53]. Z-VEID-fluoromethyl ketone (FMK), a specific irreversible inhibitor of caspase-6, was bought from R&D Systems. Human U87 MG and U87 MG-R glioblastoma cells were pretreated with 50 μM Z-VEID-FMK for 1 h and then exposed to 50 μM enzalutamide for 72 h. Next, caspase-6 activity, cell morphology, DNA fragmentation, and cell apoptosis were subsequently analyzed.

### 4.13. Statistical Analysis

The statistical significance of differences between the control and enzalutamide-treated groups was evaluated using Student′s *t*-test, and differences were considered statistically significant at *p* < 0.05. Differences between drug-treated groups were considered significant when the *p* value of Duncan′s multiple-range test was <0.05. Statistical analyses between groups over time were carried out by a two-way analysis of variance (ANOVA).

## 5. Conclusions

In summary, our data mining results revealed upregulation of *AR* gene expression in GBM patients compared to normal healthy individuals. Moreover, we successfully prepared TMZ-resistant U87 MG-R cells from TMZ-sensitive U87 MG cells as our experimental models. Our data showed augmented expression of *AR* mRNA in human U87 MG and U87 MG-R glioblastoma cells compared to normal human astrocytes. Treatment of human drug-sensitive and -resistant glioblastoma cells with enzalutamide concentration- and time-dependently killed cells via an apoptotic pathway. As to the mechanism, enzalutamide specifically increased caspase-9 activities, rather than those of caspase-8. Subsequently, enzalutamide increased levels of the proapoptotic Bax protein, decreased the mitochondrial membrane potential, and triggered the release of cytochrome c from mitochondria to the cytoplasm. Treatment of human U87 MG and U87 MG-R cells with enzalutamide caused cascade activations of caspases-9, -3, and -6. A loss-of-function strategy was then used to suppress caspase-6 activation using a specific peptide inhibitor. Our results showed that suppressing caspase-6 activity led to significant attenuations in enzalutamide-induced DNA fragmentation, cell apoptosis, and cell death. Therefore, enzalutamide induced apoptosis of human TMZ-sensitive and -resistant glioblastoma cells via an intrinsic Bax-mitochondrion-cytochrome c-dependent cascade activation of caspases. Enzalutamide has the potential to be a drug candidate for treating human GBM. In our upcoming study, we will determine the combined effects of enzalutamide and TMZ on killing human glioblastoma cells and the possible mechanisms. Furthermore, a translational study using an animal model of GBM is being carried out to confirm the effects of enzalutamide on suppressing the growth of glioblastomas.

## Data Availability

The data presented in this study are available on request from the corresponding author.

## Supplementary Materials

The following supporting information can be downloaded at: https://www.mdpi.com/article/10.3390/molecules27196666/s1, Figure S1. Detailed Western Blot images from Figure 2B; Figure S2. Detailed Western Blot images from Figure 4C; Figure S3. Detailed Western Blot images from Figure 5A.

## Sample Availability

A sample of enzalutamide is available from the corresponding author.
