# Supplementary material for: Enzalutamide Induces Apoptotic Insults to Human Drug-Resistant and -Sensitive Glioblastoma Cells via an Intrinsic Bax-Mitochondrion-Cytochrome C Caspase Cascade Activation Pathway
Source: Molecules. 2022 Oct 7;27(19):6666. doi: 10.3390/molecules27196666 (PMC9572438; doi:10.3390/molecules27196666)
Supplement: Supplementary file 1 [file molecules-27-06666-s001.zip › molecules-1886747-supplementary.pdf]

molecules

Supplementary Materials

Enzalutamide Induces Apoptotic Insults to Human Drug-Resistant and -Sensitive Glioblastoma Cells via an Intrinsic Bax-Mitochondrion-Cytochrome C Caspase Cascade Activation Pathway

Chia-Yu Chang [1,2,3,†], Jui-Tai Chen [4,5,†], Tso-Hsiao Chen [3,6] and Ruei-Ming Chen [3,7,8,9,10,*]

1 Department of Neurology, Chi Mei Medical Center, Tainan 71004, Taiwan
2 Center for General Education, Southern Taiwan University of Science and Technology, Tainan 71005, Taiwan
3 Cell Physiology and Molecular Image Research Center, Wan Fang Hospital, Taipei Medical University, Taipei 11696, Taiwan
4 Department of Anesthesiology, Shuang Ho Hospital, Taipei Medical University, Taipei 23561, Taiwan
5 Department of Anesthesiology, School of Medicine, College of Medicine, Taipei Medical University, Taipei 11031, Taiwan
6 Division of Nephrology, Department of Internal Medicine, Wan Fang Hospital, Taipei Medical University, Taipei 11696, Taiwan
7 Graduate Institute of Medical Sciences, College of Medicine, Taipei Medical University, Taipei 11031, Taiwan
8 International Ph.D. Program for Cell Therapy and Regeneration Medicine, College of Medicine, Taipei Medical University, Taipei 11031, Taiwan
9 Anesthesiology and Health Policy Research Center, Taipei Medical University Hospital, Taipei 11031, Taiwan
10 TMU Research Center of Cancer Translational Medicine, Taipei Medical University, Taipei 11031, Taiwan
* Correspondence: rmchen@tmu.edu.tw; Tel.: +886-2-27361661
† These authors contributed equally to this study.

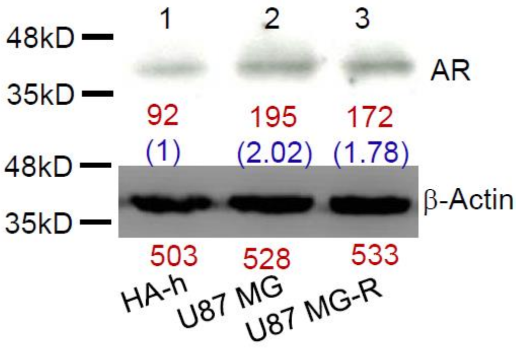

Figure S1. Detailed Western Blot images from Figure 2B.

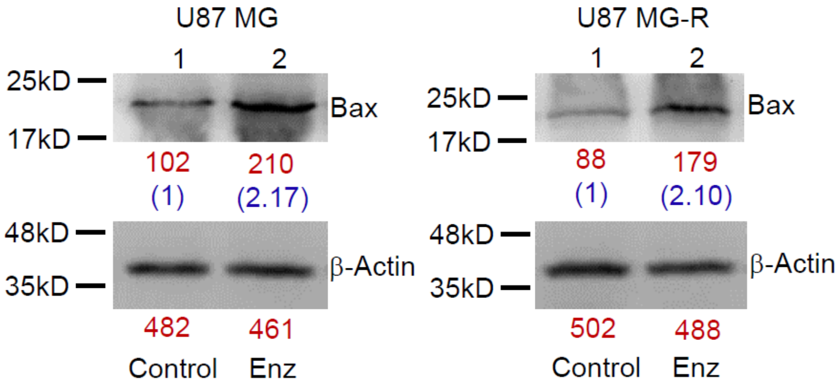

Figure S2. Detailed Western Blot images from Figure 4C.

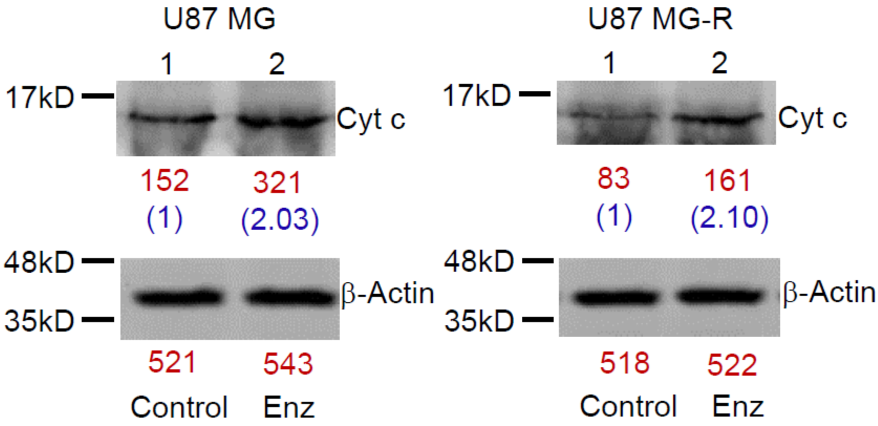

Figure S3. Detailed Western Blot images from Figure 5A.
